# Electrically Tunable Absorption Enhancement with Spectral and Polarization Selectivity through Graphene Plasmonic Light Trapping

**DOI:** 10.3390/nano6090155

**Published:** 2016-08-23

**Authors:** Wenbin Liu, Jianfa Zhang, Zhihong Zhu, Xiaodong Yuan, Shiqiao Qin

**Affiliations:** 1 College of Optoelectronic Science and Engineering, National University of Defense Technology, Changsha 410073, China; wbliu10@163.com (W.L.); zzhwcx@163.com (Z.Z.); x.d.yuan@163.com (X.Y.); sqqin8@nudt.edu.cn (S.Q.); 2 State Key Laboratory of High Performance Computing, National University of Defense Technology, Changsha 410073, China

**Keywords:** graphene, plasmonic, absorption enhancement, polarization selectivity, photodetectors

## Abstract

In this paper, anisotropic graphene plasmonic structures are explored for light trapping and absorption enhancement in surrounding media. It is shown that electrically tunable and versatile spectral and polarization selectivity can be realized. Particularly, it is possible to control absorption of the incident light’s polarization component at a specific wavelength by varying the Fermi energy with suitable geometric designs. It may find applications for new types of infrared and THz photodetectors and will promote the research of other novel polarization devices.

## 1. Introduction

Plasmonics provide a powerful platform for controlling light–matter interactions and enable a variety of novel properties for functional photonic devices. Among the many applications of plasmonics, plasmonic light trapping has been intensively studied for photovoltaics and photodetection [1,2]. Besides absorption enhancement, plasmonic structures can also provide additional advantages, such as spectral selectivity and polarization control, which can be appealing for photodetectors [3,4,5]. Doped graphene has recently emerged as a promising plasmonic material. Graphene plasmons display unprecedented spatial confinement and relatively low losses [6,7]. The excitation of graphene plasmons can significantly enhance light–matter interactions [8,9]. Moreover, the plasmons of graphene can be tuned by changing its Fermi energy with chemical or electrical doping. Recently, graphene plasmons have been proposed for light trapping and absorption enhancement in photodetectors [10]. However, the geometry and polarization properties have not been well explored. In this paper, we study the light trapping and absorption enhancement functionalities of anisotropic graphene plasmonic structures. Particularly, the electrically tunable spectral selectivity and polarization manipulation functionalities will be investigated. We will show that by combining the electrical tunability and geometric designs of graphene structures, novel polarization control properties can be realized along with plasmonic light trapping and absorption enhancement.

## 2. Results and Discussion

**Figure 1:**
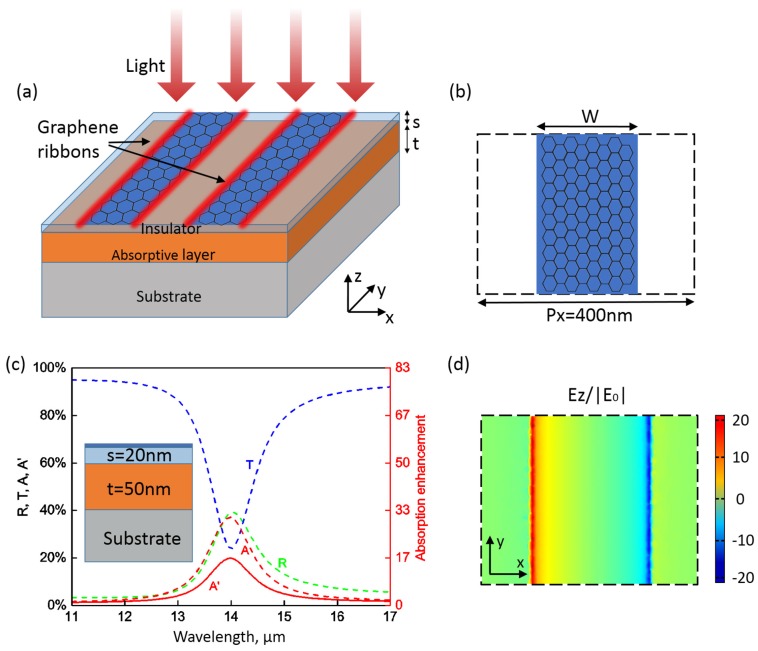
Plasmonic light trapping with graphene nanoribbon structure. (**a**) Schematic of the proposed structure. From the top to the bottom of the structure are a nanostructured graphene film with an array of periodical nanoribbons, an insulator layer with a thickness of *s*, an absorptive layer with a thickness of *t*, and a semi-infinite substrate, respectively. Incident light of transverse-magnetic (TM) (x-polarized) modes excites localized plasmons in the doped graphene layer, which trap light in the near-field and enhance the optical absorption in the light-absorbing layer underneath; (**b**) A graphene nanoribbon with geometric parameters. The period of the graphene nanoribbon is $P_{x}=400$ nm. The width is *W*; (**c**) Simulated spectra of reflection (*R*), transmission (*T*), total absorption (*A*), and absorption in the absorptive layer ($A^{\prime }$) for TM mode under normal incidence. The Fermi energy is $E_{F}=0.6$ eV. The enhancement of absorption in the absorptive layer is also shown; (**d**) Electric field distributions in the z-direction at the resonance wavelength of 14.0μm. The field is normalized to the field amplitude of the incident light ($E_{0}$) and plotted in the x–y plane that is 5 nm above the graphene nanoribbons.

We can start with a structure as shown in Figure 1a,b. Both the substrate and the insulator layer are assumed to be lossless with a dielectric constant of 1.96 (typical materials could be silicon dioxide in the far-infrared and terahertz (THz) ranges or calcium fluoride in the mid-infrared range (*n* = 1.4)). Here the insulator layer is employed for electrical insulation between the graphene and the light-absorbing material. The dielectric constant (real part) of the light-absorbing material is $\epsilon^{\prime }=10.9$, and the losses are introduced through the imaginary part $\epsilon^{\prime \prime }$ of the dielectric constant (typical materials for photodetection in mid-infrared or THz ranges could be HgCdTe (Mercury cadmium telluride, i.e., MCT) [11]).

A fully three-dimensional finite element technique (in Comsol MultiPhysics) is employed for numerical simulations [10]. In the simulations, the graphene is modelled as a conductive surface [12,13,14] and the optical conductivity of graphene can be derived within the random-phase approximation (RPA) in the local limit [15,16].

(1) $$\begin{array}{cc} \sigma_{\omega }= & \frac{2e^{2}k_{B}T}{\pi \hbar^{2}}\frac{i}{\omega +i\tau^{-1}}\ln\left[2\cosh\left(\frac{E_{F}}{2k_{B}T}\right)\right]+\\ & \frac{e^{2}}{4\hbar }[\frac{1}{2}+\frac{1}{\pi }arctan\left(\frac{\hbar \omega -2E_{F}}{2k_{B}T}\right)-\\ & \frac{i}{2\pi }\ln\frac{{\left(\hbar \omega +2E_{F}\right)}^{2}}{{\left(\hbar \omega -2E_{F}\right)}^{2}+4{\left(k_{B}T\right)}^{2}}] \end{array}$$

where $k_{B}$ is the Boltzmann constant, *T* is the temperature, *ω* is the frequency of light, *τ* is the carrier relaxation lifetime, and $E_{F}$ is the Fermi energy of graphene. We only consider highly-doped graphene with the Fermi energy $E_{F}\gg k_{B}T$ and $E_{F}\gg \hbar \omega$, so Equation (1) reduces to the Drude model [17,18].

(2) $$\sigma_{\omega }=\frac{e^{2}E_{F}}{\pi \hbar^{2}}\frac{i}{\omega +i\tau^{-1}}$$

$\tau =\mu E_{F}/\left(ev_{F}^{2}\right)$, where $v_{F}\approx 1\times 10^{6}$ m/s is the Fermi velocity and *μ* is the DC mobility. We use a moderate mobility $\mu =10,000{\mathrm{cm}}^{2}\cdot V^{-1}\cdot s^{-1}$. At first, the Fermi energy of graphene is assumed to be $E_{F}=0.6$ eV, which may be realized by electrostatic doping [19].

When the electric field of incident light is perpendicular to the graphene ribbons (x-polarized, i.e., transverse-magnetic (TM) mode), a plasmonic resonance can be exited in the studied spectral range (Here we only consider the fundamental mode, and higher order modes at shorter wavelength outside of the studied spectral range are not considered). Figure 1c shows the numerically simulated spectra under the illumination of a TM plane wave at normal incidence. Here the thickness of the insulator layer is s=20 nm. The light-absorbing layer is t=50 nm thick with an absorption coefficient $\alpha =-0.05\;{\mu m}^{-1}$ corresponding to a small absorption of only about 1.2% in impedance-matched media. The width of the graphene nanoribbon is W=200 nm. There is a resonance at around 14.0μm in the spectra with strong light extinction. The oscillation of localized surface plasmons leads to light trapping and local field enhancement around the graphene nanoribbon and serves to enhance absorption in the nearby absorptive layer. The total absorption is A=40.3%, while the absorbance by the absorptive layer reaches $A^{\prime }=21.5\%$, representing an enhancement of about 17.9 times. On the other hand, y-polarized light ( transverse-electric (TE) mode) cannot excite the localized plasmonic resonance.

**Figure 2:**
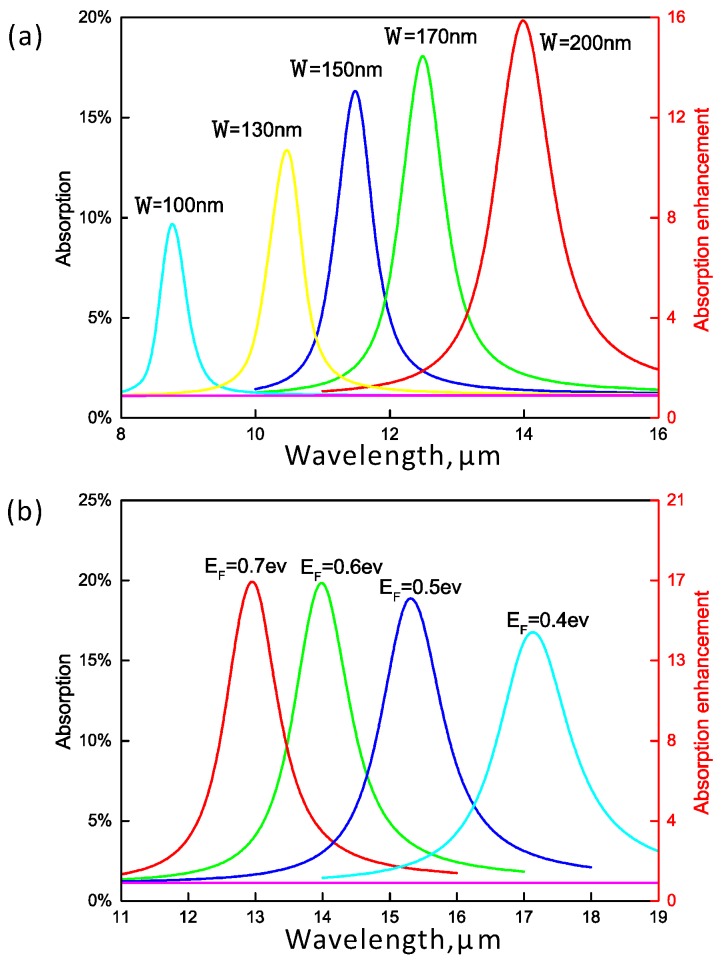
Simulated spectra of absorption in the underlying absorptive layer with different widths of (**a**) graphene ribbons; and (**b**) Fermi energies for TM mode.The enhancement factor of absorption in the absorptive layer is compared to that in an impedance matched medium. The absorption for TE (y-polarized) mode is also shown (the flat line), which almost keeps constant with the variation of the width and Fermi energy of graphene.

Figure 2a displays the spectra of absorption in the absorptive layer with the variation of the nanoribbon width. With the decrease of the width, the resonance wavelength becomes shorter. The resonance wavelength blue shifts from 14.0μm to 8.75μm when the width of graphene nanoribbon changes from 200 nm to 100 nm. We then fix the width to be W=200 nm and study the spectra of absorption in the absorptive layer with different Fermi energies of graphene between 0.4 and 0.7 eV. The spectra are shown in Figure 2b. As the Fermi energy increases, the resonance blue shifts to shorter wavelengths, and the resonant absorption goes up. Meanwhile, the conductivity of graphene increases and the graphene plasmon becomes less lossy.

The dependence of resonance wavelength on the width and Fermi energies can be explained by the following theoretical analysis. The absorption maximum wavelength corresponds to the first order graphene plasmon resonance, which occurs at

(3) $$W=\left(1-\phi /\pi \right)\lambda_{eff}/2$$

where *ϕ* is the phase of the reflection coefficient for plasmon resonance reflection at the ribbon terminations and $\lambda_{eff}$ is the effective resonance wavelength. $\lambda_{eff}=\lambda_{0}/Re\left(n_{eff}\right)$, where $\lambda_{0}$ is the vacuum wavelength of light and $Re(n_{eff})$ is the real part of effective refractive index of graphene plasmons. In the studied spectral range, the intraband response dominates the conductance, so $Re\left(n_{eff}\right)\approx \hbar \omega /\left(2\alpha_{0}E_{F}\right)$, where $\alpha_{0}$ is the fine-structure constant [20]. So, the plasmonic resonance wavelength (for the TM mode) approximately satisfies [21,22]

(4) $$\lambda_{0}=\sqrt{\left(2\pi c\hbar W\right)/(\alpha_{0}E_{F}\left(1-\phi /\pi \right))}\propto \sqrt{W/E_{F}}$$

where *c* is the speed of light in a vacuum, ℏ is the reduced Plank’s constant, and *W* is the width of graphene nanoribbons.

**Figure 3:**
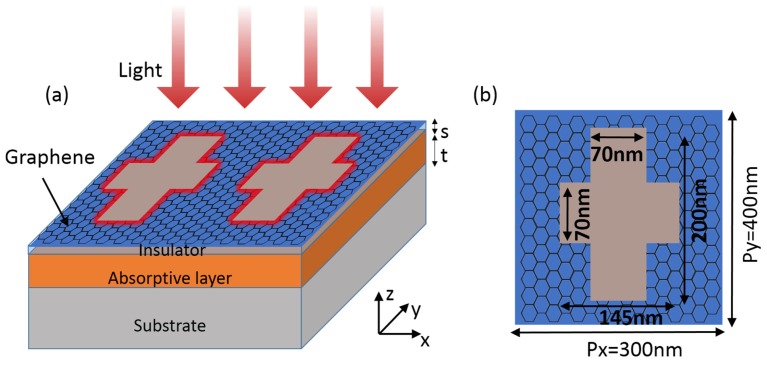
Anisotropic absorption enhancement. (**a**) The thickness of the insulator layer is s=20 nm, and the thickness of the light-absorbing layer is t=50 nm; (**b**) A unit cell of the cruciform. The period is $P_{x}=300$ nm, $P_{y}=400$ nm. The length and width of the long rectangle is 200 nm and 70 nm. The length and width of the short rectangle is 145 nm and 70 nm.

**Figure 4:**
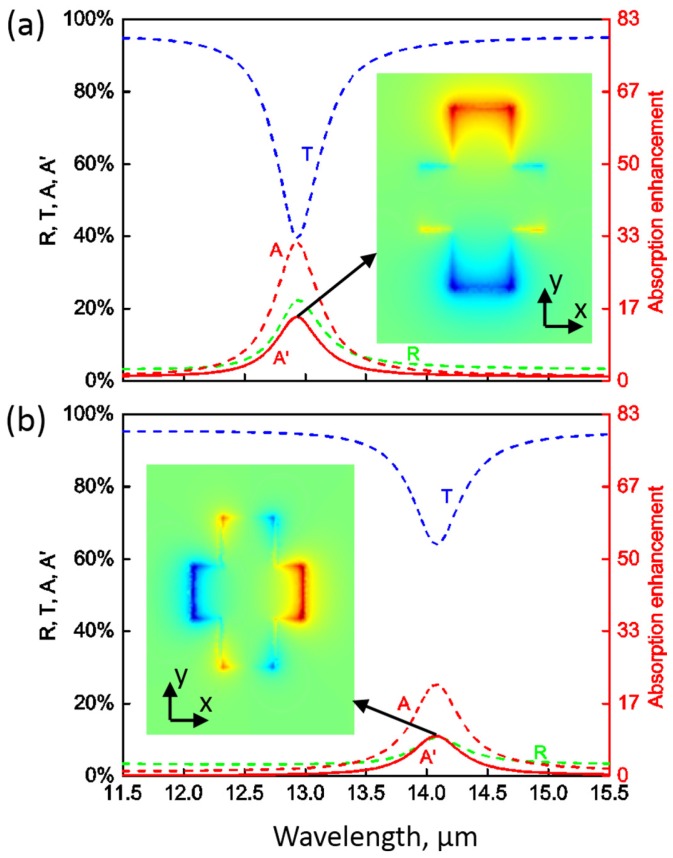
Simulated spectra of reflection (*R*), transmission (*T*), absorption (*A*) as well as absorption in the absorptive layer ($A^{\prime }$) for linearly polarized light. (**a**) For TM (x-polarized) mode at normal incidence, there is a resonance at around 12.93μm; (**b**) For TE (y-polarized) mode at normal incidence, there is a resonance at around 14.08μm. The enhancement of absorption in the absorptive layer and the magnetic field in the z-direction is also shown. The insets in (**a**,**b**) show the magnetic fields in the z-direction that are plotted in the x–y plane that is 5 nm above the graphene cross at the resonances. The Fermi energy here is $E_{F}=0.6$ eV.

In order to achieve absorption enhancement for both TM (x-polarized) and TE (y-polarized) modes, we consider the structure shown in Figure 3. Here the doped graphene film is patterned with an array of periodical anisotropic crosses. The thickness of the underlying insulator and absorptive layers, as well as their optical constants, are the same as in Figure 1. The dimensions of the crosses are shown in Figure 3b. Figure 4a,b show the numerically-simulated spectra under the illumination of TM and TE plane waves at normal incidence, respectively. Here the Fermi energy is fixed at $E_{F}=0.6$ eV. For TM mode, there is a resonance at around 12.93μm. The resonant absorption in the underlying absorptive layer reaches 17.7% with an enhancement of about 14.75. For TE mode, the resonance happens at around 14.08μm and the maximum resonant absorption in the absorptive layer is 14.2%, representing an enhancement of about 11.8 times. As we can see from the field distributions (insets in Figure 4a,b), these two excited plasmonic resonances are similar. However, the resonance wavelengths are different due to the different dimensions of the crosses in the x- and y-directions.

**Figure 5:**
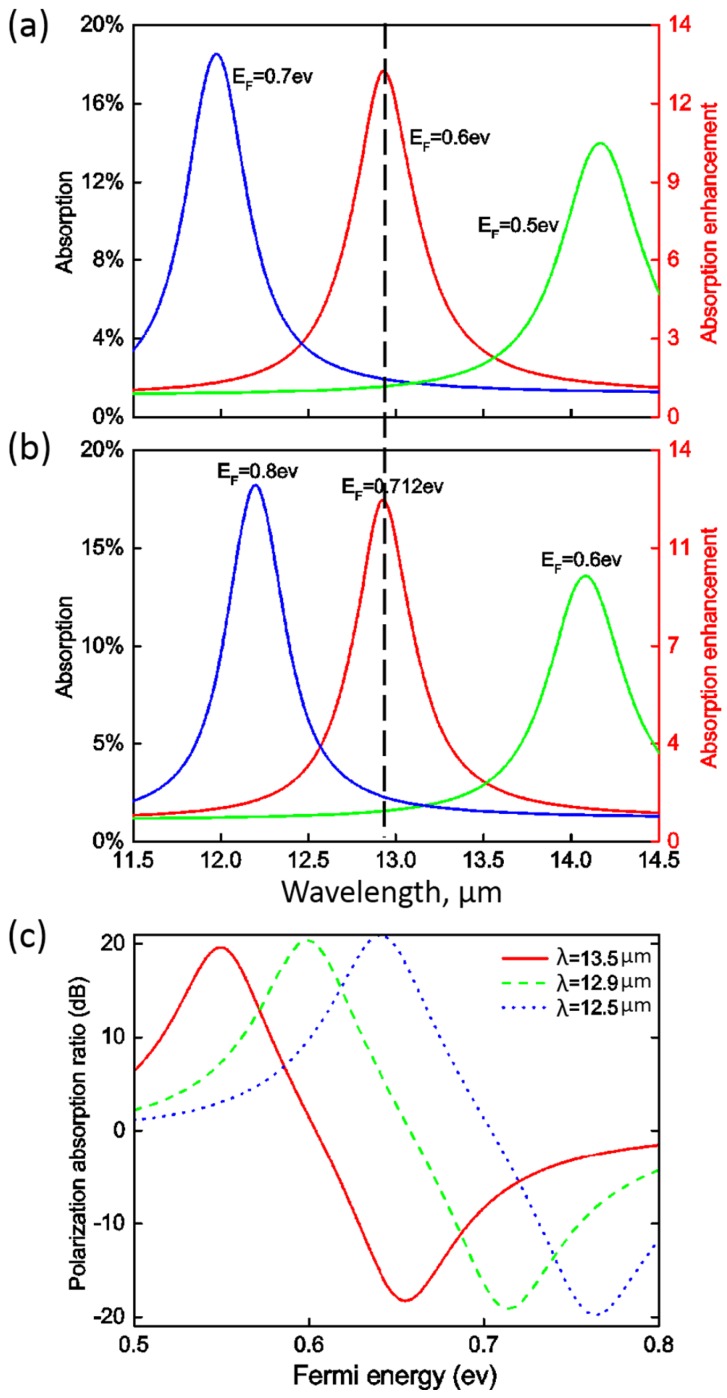
Absorption enhancement with controllable spectral and polarization selectivity. (**a**) The absorption in the absorptive layer with the Fermi energy of graphene ranging from 0.5 to 0.7 eV under the illumination of a TM mode at normal incidence is shown; (**b**) The absorption in the absorptive layer with the Fermi energy of graphene ranging from 0.6 to 0.8 eV under the illumination of TE mode at normal incidence is shown; (**c**) Electrically tunable polarization selectivity of absorption. The red, blue, and green curves show the dependence of polarization absorption ratio (defined as 20log$\left(A_{TM}^{\prime }/A_{TE}^{\prime }\right)$) on the variation of graphene’s Fermi energy at 13.5μm, 12.9μm, and 12.5μm, respectively.

Figure 5a,b shows the spectra of absorption in the absorptive layer for TM and TE modes when the Fermi energy varies. Similar to the graphene nanoribbon structures, the resonance blue shifts to shorter wavelength with the increase of graphene’s Fermi energy. Interestingly, the resonance wavelength of TM mode is 12.9μm at the Fermi energy of $E_{F}=0.6$ eV, which is the same as that of TE mode at the Fermi energy of $E_{F}=0.712$ eV. So, it is possible to absorb either TM or TE mode at the specific wavelength simply by changing Fermi energy of the graphene. Figure 5c shows the dependence of the polarization absorption ratio on graphene’s Fermi energy, which is defined as 20log$\left(A_{TM}^{\prime }/A_{TE}^{\prime }\right)$ for three different wavelengths. Here $A_{TM}^{\prime }$ and $A_{TE}^{\prime }$ are the absorption of the TM and TE modes. At the wavelength of 12.9μm, the polarization absorption ratios are about 21.0 and −19.4 dB for the Fermi energies of 0.6 eV and 0.712 eV, and the absorption ratio for TM and TE mode varies continuously with the variation of Fermi energy. Similar behavior was also observed for the other two wavelengths. This can be explored for novel infrared or THz photodetectors with tunable polarization selectivity.

## 3. Conclusions

In summary, we numerically studied the anisotropic graphene plasmonic structures for light trapping and absorption enhancement in the mid-infrared range. Tunable spectral selectivity can be achieved with localized plasmonic resonances, while polarization-dependent absorption enhancement can be realized through geometric designs of graphene nanostructures. Moreover, it is possible to control absorption of the incident light’s polarization component at the specific wavelength by varying the Fermi energy of the incident light. As such, graphene plasmonic structures make the development of a new generation of versatile infrared and THz photodetectors with high detectivity, tunable spectral selectivity, and polarization manipulating capabilities possible.

The work will also promote the research of other novel polarization devices [22,23].
